# Herbal medicine use and predictors among pregnant women attending antenatal care in Ethiopia: a systematic review and meta-analysis

**DOI:** 10.1186/s12884-020-2856-8

**Published:** 2020-03-12

**Authors:** Fentahun Adane, Girma Seyoum, Yoseph Merkeb Alamneh, Worku Abie, Melaku Desta, Bihonegn Sisay

**Affiliations:** 1 Department of Anatomy, College of Health Sciences, Addis Ababa University, Addis Ababa, Ethiopia; 2 Department of Biomedical Sciences, School of Medicine, Debre Markos University, Debre Markos, Ethiopia; 3 Department of Midwifery, College of Health Science, Debre Markos University, Debre Markos, Ethiopia; 4 Traditional and modern medicine research directorate, Ethiopia Public health institution, Addis Ababa, Ethiopia

**Keywords:** Pregnant women, Herbal medicine, Antenatal care, Ethiopia

## Abstract

**Background:**

The use of herbal medicine among pregnant women is increasing in many low- and high-income countries due to their cost-effectiveness in treatment and ease of access. Research findings across Ethiopia on the prevalence and predictors of herbal medicine use among pregnant women attending antenatal care are highly variable and inconsistent. Therefore, this systematic review and meta-analysis aims to estimate the overall prevalence of the use of herbal medicine and its predictors among pregnant women attending antenatal care in Ethiopia.

**Method:**

We searched articles in Medline (PubMed), EMBASE, HINARI, Google Scholar, Science Direct, Cochrane Library, and other sources. The study included a total of eight studies that reported the prevalence and predictors of herbal medicine use among pregnant women from different regions of Ethiopia. Cochrane Q test statistics and I^2^ tests were used to assess heterogeneity. A random effect meta-analysis model was used to estimate the pooled prevalence. In addition, the association between risk factors and herbal medicine use in pregnant women attending antenatal care were examined.

**Results:**

A total of eight studies were included in this review. The pooled prevalence of herbal medicine use among pregnant women attending antenatal care in Ethiopia was 47.77% (95% CI: 28.00–67.55). Subgroup analysis by geographic regions has showed that the highest prevalence (57.49%;95% CI: 53.14, 61.85) was observed in Oromia Region and the lowest prevalence was observed in Addis Ababa (31.39%; 95% CI: 2.83, 79.96). The herbal medicines commonly consumed by women during pregnancy were ginger: 41.11% (95% CI: 25.90, 56.32), damakasse: 34.63% (95% CI: 17.68, 51.58), garlic: 32.98% (95% CI: 22.21, 43.76), tenaadam: 19.59% (95% CI: 7.54, 31.63) and eucalyptus: 4.71% (95% CI: 1.1, 8.26). Mothers’ previous history of self-medication (95% CI: 1.91, 51.35), illness during pregnancy (95% CI: 1.56, 23.91), employment status (95% CI: 3.89, 10.89), educational status (95% CI: 1.52, 2.68), and place of residence (95% CI: 1.86, 3.23) were predictors of herbal medicine use by women during pregnancy.

**Conclusion:**

In this study**,** about half of women attending antenatal care use herbal medicine and it is relatively high. The most commonly consumed herbal medicine during pregnancy was ginger followed by damakasse, garlic, tenaadam and eucalyptus. During pregnancy, it is not known that these most commonly consumed plant species have harmful fetal effects. However, many of the medicinal plant species are poorly studied, and it is not possible to rule out teratogenic effects. Teamwork between healthcare professionals and traditional practitioners to educate on the use of medicinal plants will encourage healthier pregnancies and better health for mothers and infants.

## Background

The World Health Organization (WHO) defines traditional medicine (TM) as health methods, techniques, knowledge, and values that include medicinal products, animals and minerals, religious therapies, manual activities and exercises used individually or in conjunction with disease management, treatment, and prevention as well as well-being [1].

Among traditional medicinal practices, the use of herbal medicinal products, defined as formulations derived from plants that claim healing benefits, is the most prominent and most widely practiced by both the wider population and pregnant women around the world [2, 3].

Pregnant women use herbal medicines for a variety of reasons such as for pregnancy-associated disorders including nausea, vomiting, and labor enhancement [4], as well as for illnesses and diseases due to pregnancy such as fatigue, respiratory and skin issues, and nutritional benefits [5]. Additionally, pregnant women use herbal medicines because of their wide availability, possibly better effectiveness relative to modern medicine, traditional and cultural beliefs in herbal medicines to cure diseases and relatively low cost of these medicines [5, 6].

The use of traditional medicine accounts for approximately 40% of all health care delivered in Asian countries such as China. In Africa, traditional medicine is used by up to 80% of the population to help meet their health related needs [7]. The use of herbal medicine in Africa is correlated with lower educational levels, use before index birth, age, low socio-economic status, and trimester pregnancy [8, 9].

The most common herbal remedies consumed by pregnant women globally are ginger (*Zingiber officinale*), garlic (*Allium sativum*), green tea (*Camellia sinensis*), peppermint (Mentha piperita), and fenugreek (Trigonella foenum graecum) [10]. Studies in Australia and Kenya have shown that those pregnant women who are most likely to use herbal medicines include older and married women with low economic and educational status, nausea, and vomiting severity [8, 11]. While pregnant women’s increased use of herbal medicines worldwide, most of them are unaware of the possible side effects and teratogenic effects of some herbal remedies [8, 12]. Pregnant and breastfeeding women are especially vulnerable to harmful effects from herbal medicines as the safety profiles and appropriate dosages of most herbal medicines in these groups are not well established [13].

Approximately 80% of the population in Ethiopia use traditional medicine [14]. Traditional medicine, including the use of herbal medicines in Ethiopia, is not only common but also culturally accepted [15]. Studies have found that most districts in Ethiopia have an inconsistent prevalence of herbal medicine use in pregnant mothers with antenatal care (ANC), ranging from 10.9 to 73.1% [16, 17]. Furthermore, the risk factors associated with herbal medicine use among pregnant mothers and the type of herbal medicine they use differ from district to district. While there is insufficient evidence in this area, the study results are variable, and therefore it is prudent to work towards synthesizing the evidence. The aim of this systematic review and meta-analysis was to estimate the pooled prevalence of herbal medicine use among pregnant women attending ANC in Ethiopia, and to identify the predictors that are associated with herbal medicine use among pregnant mothers attending ANC in Ethiopia. The findings of this meta-analysis will help policy makers and other stakeholders in planning and implementing strategies to create awareness about potential side-effects of traditional medicine on the fetus; some herbal products may be teratogenic for humans [14]. The analysis could also be used as a basis for performing confirmatory investigations by investigators. The review question posed was: What are the pooled prevalence and predictors for herbal medicine use among pregnant women attending ANC care in Ethiopia?

## Methods

### Identification and study selection

Three authors (FA, GS & YM) have identified both published articles and unpublished researches reporting the prevalence and predictors of herbal medicine use among pregnant mothers attending ANC care in Ethiopia. Studies were identified through a literature search of Medline (Pub Med), EMBASE, HINARI, Google Scholar, Science Direct, Cochrane Library, and other sources. The reference list for each included article was also manually searched for search optimization. The search was conducted from January 9, 2019 to October 15, 2019, and it was limited to English language. Unpublished studies have also been searched through Google and Google Scholar. The search terms were predetermined for an extensive search that included all fields in records, as well as Medical Subject Headings (MeSH terms) to expand the search in an advanced PubMed search. We merged keywords with the “OR” in the Boolean operator within each axis and then related the search strategies for the two axes to the “AND” operator. The key terms used for the search were “Prevalence” OR “Epidemiology” AND “Traditional Medicine” AND/OR “Herbal Medicine” OR “Pregnant Mothers” AND/OR “Pregnant Women” OR “antenatal follow up” AND “Ethiopia”. The specific searching detail in pubmed with MeSH terms was (“herbal medicine” [MeSH Terms] OR (“herbal” [All Fields] AND “medicine” [All Fields]) OR “herbal medicine” [All Fields]) AND (“gravidity” [MeSH Terms] OR “gravidity” [All Fields] OR “pregnant” [All Fields]) AND (“mothers” [MeSH Terms] OR “mothers” [All Fields]) AND (“Ethiopia” [MeSH Terms] OR “Ethiopia” [All Fields]) were used. All literatures accessible until October 2019 were included in the systematic review and meta-analysis. The systematic review and meta-analysis was carried out in accordance with the Preferred Reporting Items for Systematic reviews and Meta-Analyses (PRISMA) guidelines [18].

### Eligibility criteria

#### Inclusion criteria

Articles included were those reporting the prevalence of herbal medicine use and predictors among pregnant mothers attending ANC care in Ethiopia.

##### Study area

Only articles conducted in Ethiopia.

##### Study design

All observational studies (cross-sectional, case controls, and cohort) that contain original data reporting the prevalence and predictors of herbal medicine use among pregnant mothers attending ANC in Ethiopia were considered.

##### Language

Literatures written in English language.

##### Population

Studies that have been considered among pregnant mothers in ANC in Ethiopia.

##### Publication condition

A consideration was given to both published articles and unpublished research.

#### Exclusion criteria

Non-accessible researches which are unpublished, irretrievable from the internet or failed to receive replies to email from corresponding authors were excluded. In addition, research that did not report our outcome of interest was excluded after reviewing complete texts (by three authors (FA, GS and YM).

### Data abstraction

All the data required were extracted using a clear data extraction format prepared by two authors (FA and MD) in Microsoft Excel™. For the prevalence of herbal medicine use, the data extraction format prepared based on first author, the region where the study was carried out, publication year, sample size, and prevalence of herbal medicine use stated for the target group.

For predictors, the data extraction format was prepared for each specific predictor (maternal residence, maternal Educational status, maternal occupation, maternal illness and previous self-medication). The researchers selected these variables because they are the most commonly reported associated risk factors in the studies included in this meta-analysis. In this systematic review and meta-analysis, the investigators considered additional variables as risk factors if two or more studies investigated them as risk factors. For every associated risk factor, to compute the odds ratio, the data from the primary studies were extracted in the form of two by two tables by three authors (FA, GS and BS).

### Outcome measurements

This systematic review and meta-analysis have two main outcomes. The primary outcome was the prevalence of herbal medicine use among pregnant women attending ANC in Ethiopia. The second outcome of the study was predictor of herbal medicine use among pregnant women attending ANC in Ethiopia. The prevalence was computed by dividing the number of pregnant mothers attending ANC who use herbal medicine by the total number of pregnant mothers attending ANC in the study (sample size) multiplied by 100.

### Quality assessment

The researchers (FA & YM) used the Newcastle-Ottawa Scale adjusted for the quality evaluation of the cross-sectional studies to determine the quality of the studies included in this review [19]. The tool consists of three basic parts; the first section has five stars, and assesses each study’s methodological excellence. The second part of the instrument tests the research comparability and gives two points. The last component measures the consistency of the original articles with respect to their statistical analysis and can be rated out of 3 stars. The qualities of each of the original articles were measured using the tool as a checklist. Articles included in this study have medium to high quality scores (6 out of 10 stars).

### Statistical analysis

The necessary data were extracted using a Microsoft Excel™ format and analyzed using the program STATA Version 15.0. The original studies were described using forest plots and tables. The researchers determined the standard error prevalence by the binomial distribution method for each original article. Heterogeneity among the recorded prevalence of studies has been confirmed by the use of test heterogeneity x^2^, I^2^ and *p*-values [20]. The above statistical analyses suggested a considerable heterogeneity among the studies (I^2^ = 99.2%, *p*-value < 0.001). As a result, a random effect meta-analysis model was conducted to estimate the combined effect of Der Simonian and Laird. Additionally, univariate meta-regression model was performed by taking year of publication and sample size to identify the likely source of heterogeneity, but none was found to be statistically significant. Possible publication bias was tested objectively using Egger’s correlation and Begg’s regression intercept tests at 5% significant level, respectively [19, 21]. The Egger’s weighted regression and Begg’s rank correlation test methods were also used to assess publication bias (*P* > 0.05), revealing statistically insignificant publication bias. In addition, to reduce the random differences between the primary study’s point estimates, subgroup analysis was performed based on the area in which the studies were conducted.

## Results

### Search results

**Fig. 1:**
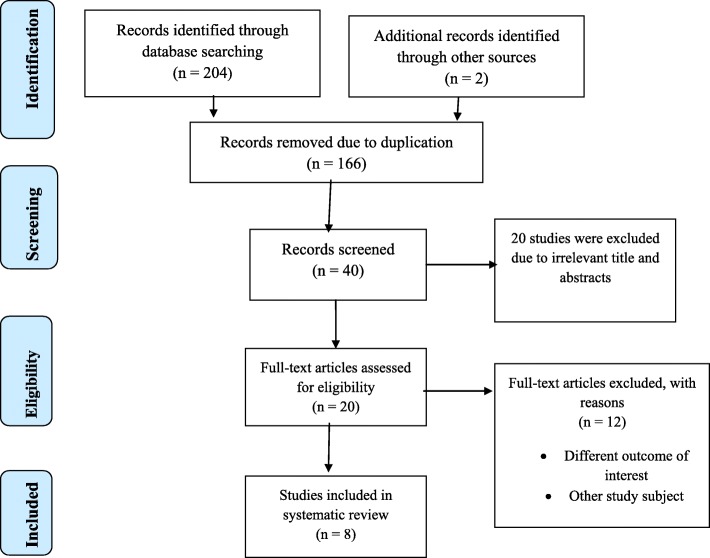
Flow chart describing the selection of studies for the systematic review and meta-analysis of prevalence and use of herbal medicine predictors among pregnant women attending antenatal care in Ethiopia (showing how articles were identified, screened, and included in the studies)

A total of 206 articles regarding the prevalence and predictors of herbal medicine use among pregnant mothers attending ANC in Ethiopia were retrieved by searching the following databases: Medline (Pub Med), EMBASE, HINARI, Google Scholar, Science Direct, Cochrane Library and other sources described above. Among these preliminary records, 166 articles were removed due to duplication. From the remaining 40 articles, 20 articles were excluded as they were found to be non-applicable after assessing their titles and abstracts. The remaining 20 full-text articles were then accessed, and measured for eligibility based on the preset standards, which resulted in the further exclusion of 12 articles primarily due to non-eligibility of the study population and outcome of interest. Four of these studies have been carried out in countries other than Ethiopia: Uganda [22], Nigeria [23], Kenia [8] and South Africa [24]. The remaining eight studies were conducted in various regions of Ethiopia [25–32] and were excluded due to the study population and unreported outcome of interest. The quality scores of each research evaluated ranged from 7 to 9 out of 10 points; thus, no papers were removed by this criterion. Finally, 8 studies were included in the final meta-analysis (Fig. 1).

### Characteristics of original articles

A total of 8 original studies that showed the prevalence and predictors of herbal medicine use among pregnant women attending ANC in Ethiopia were included in this systematic review and meta-analysis. The studies were conducted from 2009 to 2018. The study designs for all included research were cross-sectional studies. In this study, 2608 study participants were involved to estimate the pooled prevalence of herbal medicine use and its predictors among pregnant women attending ANC in Ethiopia.

The studies were conducted in Oromia Region [15, 33], Southern Nations Nationalities and Peoples’ (SNNP) Region [17], Amhara Region [34, 35], and Addis Ababa [16, 36, 37]. The sample sizes ranged from 52 in the Addis Ababa [37] to 617 in another study conducted in Addis Ababa [16] (Table 1).

**Table 1:** Descriptive summary of 8 studies reporting the prevalence of herbal medicine use among pregnant women attending ANC in Ethiopia included in the systematic review and meta-analysis

Author	Publication Year	Region	Sample Size	Case	Quality score (10 pts)	Prevalence with 95%
Mekuria et al. [34]	2017	Amhara	364	177	9	48.60 (43.47, 53.73)
Laelago et al. [17]	2016	SNNP	353	258	9	73.10 (68.47, 77.73)
Bayisa et al. [15]	2014	Oromia	250	142	8	56.80 (50.66, 62.94)
Abeje et al. [35]	2015	Amhara	128	27	8	21.10 (14.03, 28.17)
Kebede et al.(37)48	2009	Addis Ababa	52	28	8	53.80 (40.25, 67.35)
Nega et al. [36]	2019	Addis Ababa	600	360	8	60.00 (56.08, 63.92)
Jambo et al. [33]	2018	Oromia	244	142	7	58.20 (52.01, 64.39)
Beza [16]	2018	Addis Ababa	617	67	9	10.90 (8.44, 13.36)

### Meta-analysis

#### The prevalence of herbal medicine use among pregnant women attending ANC in Ethiopia

**Fig. 2:**
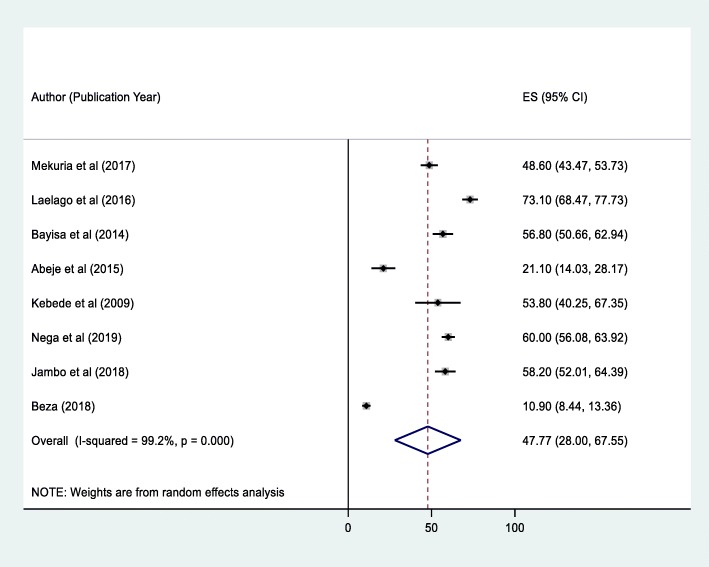
Forest plot of the pooled prevalence of herbal medicine use among pregnant women attending antenatal care in Ethiopia, 2019

Eight studies in Ethiopia showed that the pooled prevalence of herbal medicine use by pregnant mothers attending ANC was 47.77% (95% CI: 28.00–67.55) (Fig. 2). However, considerable heterogeneity was found across the studies as revealed by I^2^ statistic (I^2^ = 99.2%, *p*-value *<* 0.005). Therefore, a random effect model was used to estimate the pooled prevalence of herbal medicine use among pregnant women attending ANC in Ethiopia. A univariate meta-regression model was also carried out to identify the possible sources of heterogeneity, by considering factors, such as publication year and sample size. However, none of these variables was found to be statistically significant. Beggs’ and Eggers’ tests also revealed the absence of statistically significant publication bias, *p* > 0.05.

### Sub- group analysis

**Fig. 3:**
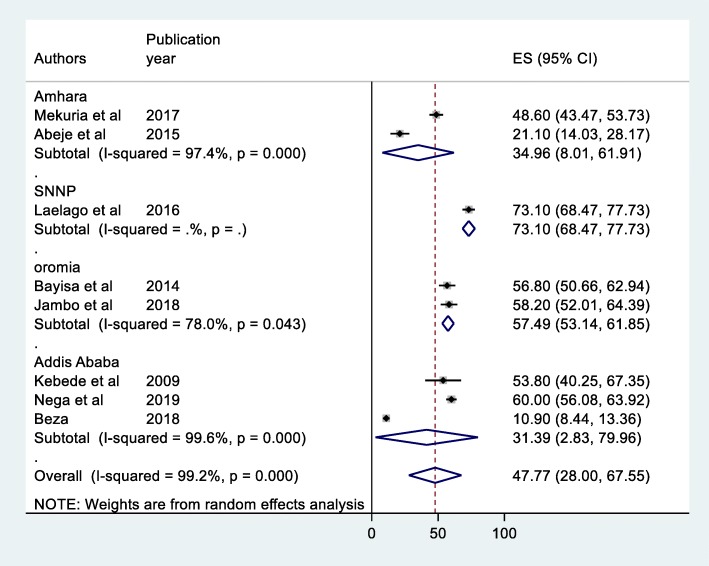
Forest plot of the Sub - group analysis of prevalence of herbal medicine use among women pregnant women attending ante-natal care in Ethiopia, 2019

Due to significant heterogeneity among the articles included in this study, region-based sub-group analysis was conducted to identify the potential source of heterogeneity in the studies. Although only one study in Southern Nations Nationalities and Peoples Region prevalence was found to be 73.1% (95% CI: 68.67–77.73), sub- group analysis shows the highest prevalence was observed in Oromia Region with prevalence of 57.49% (95% CI: 53.14–61.85) followed by a Amhara region 34.96% (95% CI: 8.01–61.91) while the lowest prevalence was observed in Addis Ababa 41.39% (95% CI: 2.83–79.96) (Fig. 3).

#### Types of herbal medicine used by pregnant women attending ANC in Ethiopia

**Fig. 4:**
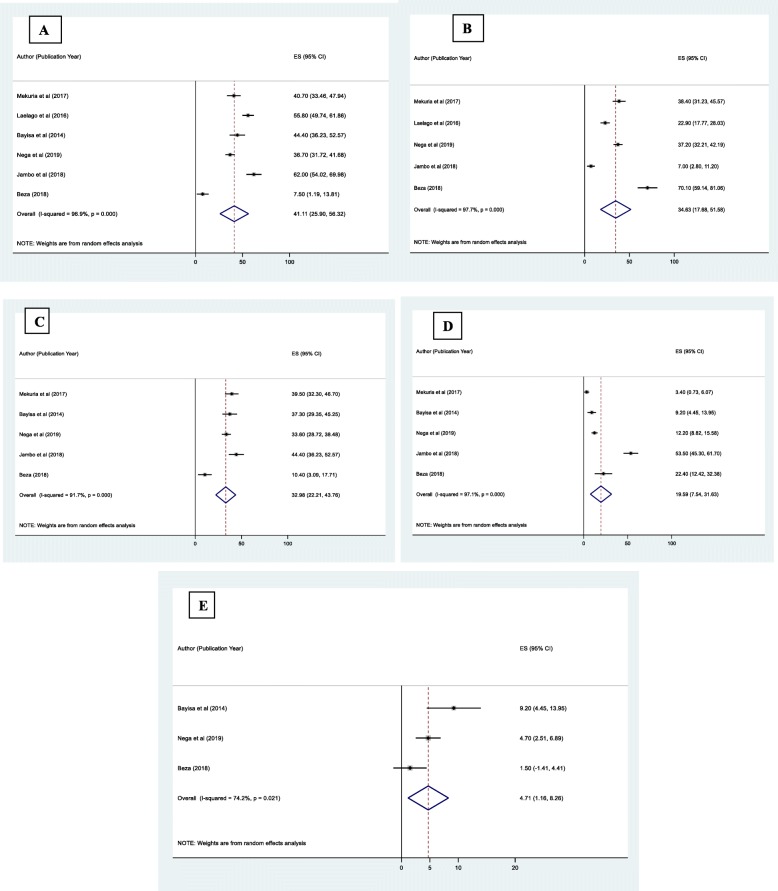
Forest plot depicting the pooled prevalence of types of herbal plants used by pregnant women; (**a**: Ginger, **b**: Demakasse, **c**: Garlic, **d**: Tenaadam, **e**: Eucalyptus), in Ethiopia, 2019

Herbal medicines commonly taken by women during pregnancy were ginger (*Zingiber officinale*) (41.11%; 95% CI:25.90, 56.32), damakasse (*Ocimum lamiifolium*) (34.63%; 95% CI:17.68, 51.58), Garlic (*Allium sativum*) (32.98%; 95% CI:22.21, 43.76), Tenaadam (*Rutachalepensis*) (19.59%; 95% CI:7.54, 31.63) and Eucalyptus (4.71%; 95% CI:1.16, 8.26) (Fig. 4a-e).

#### Predictors of herbal medicine use among pregnant women attending ANC in Ethiopia

Maternal previous history of self-medication (95% CI: 1.91, 51.35), Maternal illness during pregnancy (95% CI: 1.56, 23.91), Maternal occupation (95% CI: 3.89, 10.89), Maternal education (95% CI: 1.52, 2.68) and maternal residency (95%CI: 1.86, 3.23) were significantly associated with herbal medicine use among women during pregnancy.

**Fig. 5:**
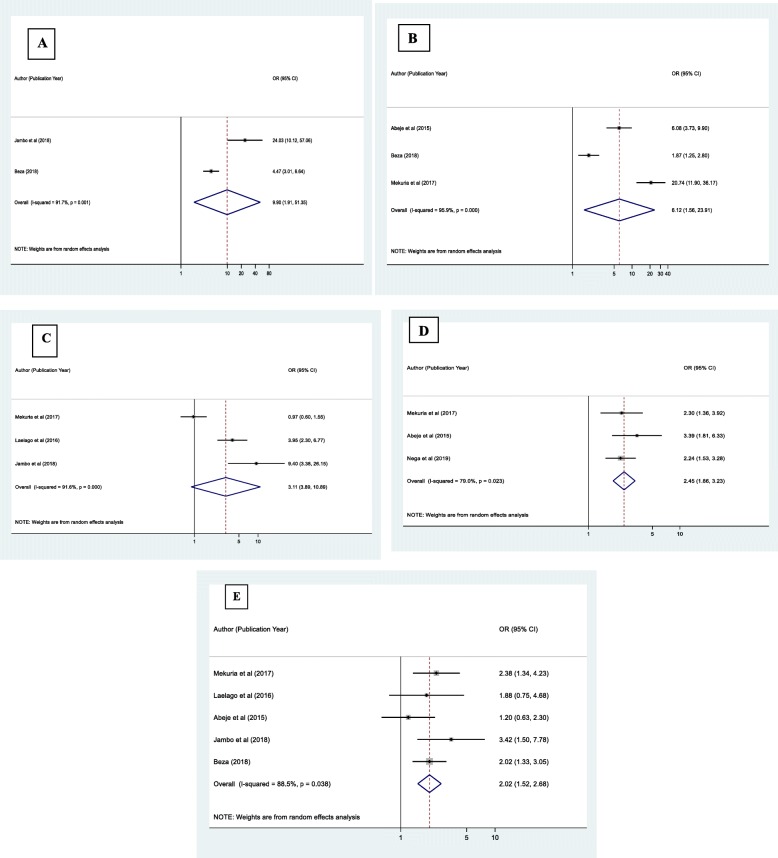
Forest plot depicting pooled odds ratio (log scale) of the associations between prevalence of herbal medicine use among pregnant women’s and its predictors (**a**: Maternal Previous self-medication **b**: maternal illness **c**: maternal occupation, **d**: maternal Residency, **e**; Maternal education), in Ethiopia, 2019

In this study, the probability of herbal medicine use among pregnant mothers who have previous history of self-medication was about 9.90 times higher than mothers who have no history of self-medication (OR = 9.90 [95% CI: 1.91,51.35]). Also, pregnant mothers who reported a history illness during pregnancy were 6.12 times more likely to use herbal medicine when compared with those who had no history of illness (OR = 6.12, [95% CI: 1.56, 23.91]). House wife pregnant women were 3.11 times more likely to use herbal medicine than those pregnant women’s who had occupation (OR = 3.11, [95% CI: 3.89,10.89]).The odds of herbal medicine use during pregnancy was 2.45 times higher among rural residents as compared to urban residents (OR = 2.45, [95%CI: 1.86, 3.23]). Finally, pregnant women who were illiterate (no formal education) were 2.02 times more likely to use herbal medicine than those who attended more than primary school education (OR = 2.02, [95% CI: 1.52,2.68]) (Fig. 5a-e).

## Discussion

In this study, an attempt was made to determine the pooled prevalence of herbal medicine use and predictors among pregnant women who attend antenatal clinics in Ethiopia. The study found that the overall prevalence of herbal medicine use in Ethiopia among pregnant women attending ANC was 47.77%. This result is similar to study carried out in Malaysia [38], which has reported a herbal medicine use magnitude of 51.4%. On the other hand, the prevalence of herbal medicine use observed in the present study is lower than the prevalence reported (68%) in a study conducted in Nigeria [39] and 85.2% prevalence reported in Malaysia [40]. However, the observed magnitude of herbal medicine use in this study was greater than the research findings in Kenya (12%) [8] and Italy (27.8%) [41]. The common practice of herbal medicine in the current study can be explained in part by the fact that tradition and culture in Ethiopia promote the use of herbal medicines which is further reinforced by the presence of many traditional medicinal practitioners [42, 43]. Differences in study design, population sampling and ethnicity may also lead to discrepancies in prevalence rates in the above studies compared to the present study.

Sub-group analysis of this study showed that the prevalence of herbal medicine use among pregnant women attending ANC varies greatly across regions. The highest prevalence of herbal medicine use was observed in Oromia Region, followed by Amhara Region while the lowest prevalence was seen in Addis Ababa. The explanation for this high prevalence of use of herbal drugs in the Oromia and Amhara region may be due to a reason that ANC health facilities for pregnant women in the Oromia and Amhara region are not as sufficient and available as they are in Addis Ababa. Since Addis Ababa is the country’s capital city, urban setting will have better healthcare facilities than the two regions mentioned above. Mother’s level of education could also be the other explanation. Herbal medicine can therefore be the only accessible health care option within a reasonable distance [31].

In our study, the herbal medicines commonly taken by women during pregnancy were ginger (*Zingiber officinale*), damakasse (*Ocimuml amiifolium*), Garlic (*Allium sativum*), Tenaadam (*Rutachalepensis*) and Eucalyptus. In line with this review, a study conducted in Alexandria found that ginger is the most frequently used herb in pregnant women [44]. However, peppermint was the most widely used herb in a Virginian study [45]. The pattern variation across different regions may be due to differences in herbal availability and geographic distribution.

In addition, the present study shows that there are significant associations between herbal medicine use and maternal previous history of self-medication, maternal illness during pregnancy, maternal occupation, maternal education and maternal residency. In this study, the probability of herbal medicine use among pregnant mothers who have previous history of self-medication was about 9.90 times higher than mothers who have no history of self-medication. This finding was in line with previous studies conducted in Democratic Republic of Congo and Nigeria [46, 47]. This may be due to the presence of chronic diseases, prior drug use, problems related to pregnancy and delivery, and low understanding of herbal drug use embryonic risks [16]. Another possible reason could be that drugs are commonly used before pregnancies give the impression that over - the-counter medicines can be used safely during pregnancy. A woman who has used moderate-illness medication also needs to take medicine for any discomfort during pregnancy [47]. They will use herbal medicine when they have no medicines in their area, or there may be no pharmacy near their home, or have no money to buy drugs.

The current study found that pregnant mothers who had a history of illness during pregnancy were 6.12 times more likely to use herbal medicine relative to those who had no history of illness. This result is comparable to other research carried out in Democratic Republic of Congo [46]. The possible reasons could be because the previous experience of using medication to treat problems related to pregnancy and childbirth effects as well as the medications used were considered safe [48].

In the present study, housewife pregnant women were 3.11 times more likely to use herbal medicine than those pregnant women who had job. The finding is in line with data from other developing countries such as India [49]. This may be because housewives are more likely to stay at home than working mothers, and they will not have any awareness about the harmful effects of modern and conventional drugs taken during pregnancy. Another possible reason is that housewives are often uneducated mothers. People without modern education, due to the influence of culture in developing countries often use traditional medicine [50].

Moreover, the odds of herbal medicine use during pregnancy was 2.45 times higher among rural residents as compared to urban residents. This finding was comparable to other research finding carried out in Nigeria [5]. This could be due to poor awareness among pregnant women in rural areas of the risk of herbal drug use during pregnancy, relatively low income and educational status among rural women, and health facilities in rural areas are not sufficient and available to pregnant women compared to urban women [15].

Finally, pregnant women who were illiterate (no formal education) were 2.02 times more likely to use herbal medicine than those who attended more than primary school education. This result is similar to study carried out in Nigeria [5], Kenya [8] and Ghana [51]. The possible justifications for this could be because of the reason that educated mothers are able to read and write; they may have better information about the effects of herbal medicine use on the fetus than uneducated mothers [52]. Another explanation for this could be due to a reason that educated mothers tend to use less traditional medicine because they do not go to a traditional medicine practitioner because their cultural outlook is changed [53].

### Strengths and limitations of the study

The strength of this meta-analysis is that it is the first of its kind in Ethiopia and it lies in the quest for existing and unpublished research and the use of multiple thoughts to strengthen the study.

However, in this systematic review and meta-analysis, all the articles are cross-sectional in design. As a consequence, temporal relationships between factors and outcome variables cannot be established. Most of the research included in this review had a small sample size that could influence the final estimate. Furthermore, since this meta-analysis included accessible research recorded from a small region in Ethiopia, the various areas in the nation may be under-represented.

## Conclusions

In this study, about half of women attending antenatal care use herbal medicine and it is relatively high. The highest prevalence of herbal medicine use among pregnant mothers was observed in Oromia Region followed by the Amhara Region where as the lowest prevalence was observed in Addis Ababa. The most commonly used herbal medicines during pregnancy were ginger followed by damakasse, Garlic, Tenaadam, and Eucalyptus. During pregnancy it is not known that the most commonly used plant species have harmful fetal effects. However, many of the medicinal plant species are poorly studied, and it is not possible to rule out teratogenic effects.

Maternal previous history of self-medication, maternal illness, maternal occupation, maternal education and maternal residency were significantly associated with herbal medicine use during pregnancy. Based on the findings, therefore, it is recommended that efforts be made to ensure that health education should be provided during ANC sessions and through media about the likely effects of using herbal medicine without a safety profile and a dose confirmation study carried out during pregnancy. In addition, hospitals should encourage traditional practitioners to work with modern practitioners and approve and communicate to the community the safety and effectiveness of herbal medicines used during pregnancy.

### Acronyms

TM (Traditional Medicine), ANC (Ante Natal Care), WHO (World Health Organization) and SNNP (Southern Nations and Nationalities of Peoples).

## Data Availability

The datasets used and/or analyzed during the current study are available from the corresponding author on reasonable request.
